# Barriers to access improved water and sanitation in poor peri-urban settlements of Abidjan, Côte d’Ivoire

**DOI:** 10.1371/journal.pone.0202928

**Published:** 2018-08-28

**Authors:** Eliachie Larissa Eméline Angoua, Kouassi Dongo, Michael R. Templeton, Jakob Zinsstag, Bassirou Bonfoh

**Affiliations:** 1 Département Recherche et Développement, Centre Suisse de Recherches Scientifiques en Côte d’Ivoire, Abidjan, Côte d’Ivoire; 2 Unité de Formation et de Recherche des Sciences de la Terre et des Ressources Minières, Université Félix Houphouët-Boigny, Abidjan, Côte d’Ivoire; 3 Department of Epidemiology and Public Health, Swiss Tropical and Public Health Institute, Basel, Switzerland; 4 University of Basel, Basel, Switzerland; 5 Department of Civil and Environmental Engineering, Imperial College London, London, United Kingdom; Mercator Research Institute on Global Commons and Climate Change gGmbH, GERMANY

## Abstract

Achieving access to safe water and sanitation still pose major challenges in urban areas of sub-Saharan Africa countries, despite all the progress achieved in the last decade. This study assessed the ability of populations living in poor peri-urban settlements to access improved water and sanitation and identified factors influencing this access, in order to guide sustainable mitigating solutions to address associated health and environmental risks. We conducted a cross-sectional study in six poor peri-urban settlements of Yopougon, the largest municipality of Abidjan. A total of 556 randomly selected households were included. The factors associated with access to improved water and sanitation were identified through explanatory models using multivariate logistic regression. A proportion of 25% of all households assessed did not have access to clean water and 57% lacked improved sanitation. Socioeconomic status and settlement characteristics appear as the main indicators of poor access to reliable water and sanitation in peri-urban settlements. The presence of the household head’s wife at home was associated with greater access to clean water (OR = 3.57; 95% CI: 1.74, 7.31), thus highlighting the important role of women in ensuring access to clean water in these specific environments. Household size, education and religion were not significantly associated with the two considered outcomes. Women therefore should be involved at all levels of programming in water promotion in these settlements to improve the population’s well-being. While religion does not appear to play an important role in access to water and sanitation, successful interventions should involve religious communities because of their large representation.

## Introduction

The concepts of water, sanitation and hygiene (WASH) initiatives are based on good hygiene practice, access to improved water supply and improved sanitation, which are essential to reduce environmental health risks for population’s well-being at the global level [1]. Worldwide, 61.1 million disability-adjusted life-years (DALYs) are attributed to the use of unimproved water (95% UI 49.4 million to 69.6 million; 85.4% of diarrheal DALYs) and 40.0 million DALYs (36.0 million to 44.4 million) to a lack of basic sanitation services [2]. Children in resource-poor areas under five years of age contribute the most towards those disability numbers. Minimizing these risk factors could prevent about 5.5% of deaths in this age group [3].

The lack of access to water and sanitation mostly affects people living in extreme poverty who are vulnerable and marginalized [4]. These are particularly slum dwellers living in precarious settlements in deprived urban areas [5] and those in rural areas or disadvantaged urban fringes [6]. In 2014, the percentage of the urban population in sub-Saharan Africa (SSA) who were living in slums was estimated at 55%. The target 7c of the Millenium Development Goals (MDG) to halve the percentage of the people without sustainable access to safe drinking water and basic sanitation services by 2015 was not achieved in any West African country [7]. In 2015, about 70% of the sub-Saharan African population were still using unimproved sanitation and 32% were relying on unimproved drinking water sources [7]. Now, the progress related to WASH is guided by the Sustainable Development Goal (SDG) targets 6.1 and 6.2 which aim to achieve, universal and equitable access to safely managed drinking water, sanitation and hygiene and end open defecation by 2030 [8].

In Côte d’Ivoire, an estimated 91.5% of the urban population can access improved drinking water, while improved sanitation facilities are only available to 31.7% [9]. However, the increasing urbanization rate of the population in the economic capital Abidjan due to rural exodus and the sociopolitical crisis faced by the country during 2002 to 2011, leads increasingly to the creation of unplanned and informal settlements. These settlements are generally excluded from access to basic urban services including sanitation, water and waste collection [10]. Indeed, people living in rural areas move into cities for a better livelihood because of the extreme poverty. In rural areas conditions of life, access of infrastructures, water and sanitation are bad compared to conditions in cities [11]. During the crisis in Côte d’Ivoire, population from the Northern, Central and Western zones, fled the war towards the southern areas of the country with a large part stayed in the city of Abidjan. Due to the deplorable economic condition, these two types of migrants have settled mainly in disadvantaged peri-urban neighborhoods. Nearly half a million people are living in such precarious settlements in and around Abidjan. Multiple studies have been undertaken in disadvantaged urban areas of Yopougon, the largest municipality of Abidjan, revealing many shortcomings in water supply and sanitation management systems, that expose people to diseases related to sanitation such as malaria, thyphoid fever and diarrhea [10, 12, 13]. Despite all the efforts to address this situation, the progress is slow. It is therefore important to understand the social, economic and demographic factors that influence access to water and sanitation in these settings, in order to find integrated and sustainable solutions toward mitigating the associated risks. Specifically, inequalities related to those factors at the household or individual level must be better understood in order to capture the most vulnerable groups concerning access to water and sanitation [9].

This study assessed the access to clean water and improved sanitation services among populations living in poor peri-urban settlements around Abidan, and investigated factors influencing this access, in order to identify sustainable mitigating solutions to avert the associated health and environmental risks.

## Methods

### Ethical considerations

Our study was approved by the institutional review board of the Centre Suisse de Recherches Scientifiques en Côte d'Ivoire (CSRS). Meetings were held with local authorities, community leaders and key informants during which the objectives, procedures and potentials risks and benefits of the study were explained. The study received a research authorization provided by the municipality of Yopougon (N0 20 / MYOP / CAB / SG-2015). Oral consent was chosen over written informed consent, owing to the high level of illiteracy in this part of the city, and was obtained from all participants. The institutional review board of the CSRS also approved the use of verbal consent in this study. Participation was voluntary and people could withdraw from the study anytime without any further obligation. All data were anonymized for analysis and reporting.

### Study area

The current study was carried out in Abidjan, located at 4°10’–5°30’N latitude and 3°50’–4°10’W longitude. It borders the Ebrié lagoon and the Atlantic Ocean in the south. The autonomous district of Abidjan has 13 municipalities, including Yopougon, which is located in the western part of Abidjan. It is the largest and most populated municipality of Abidjan with an estimated population of 1,071,543 inhabitants [14] and it covers approximately 153.06 km^2^. It contains the largest slums in the district of Abidjan.

**Fig 1:**
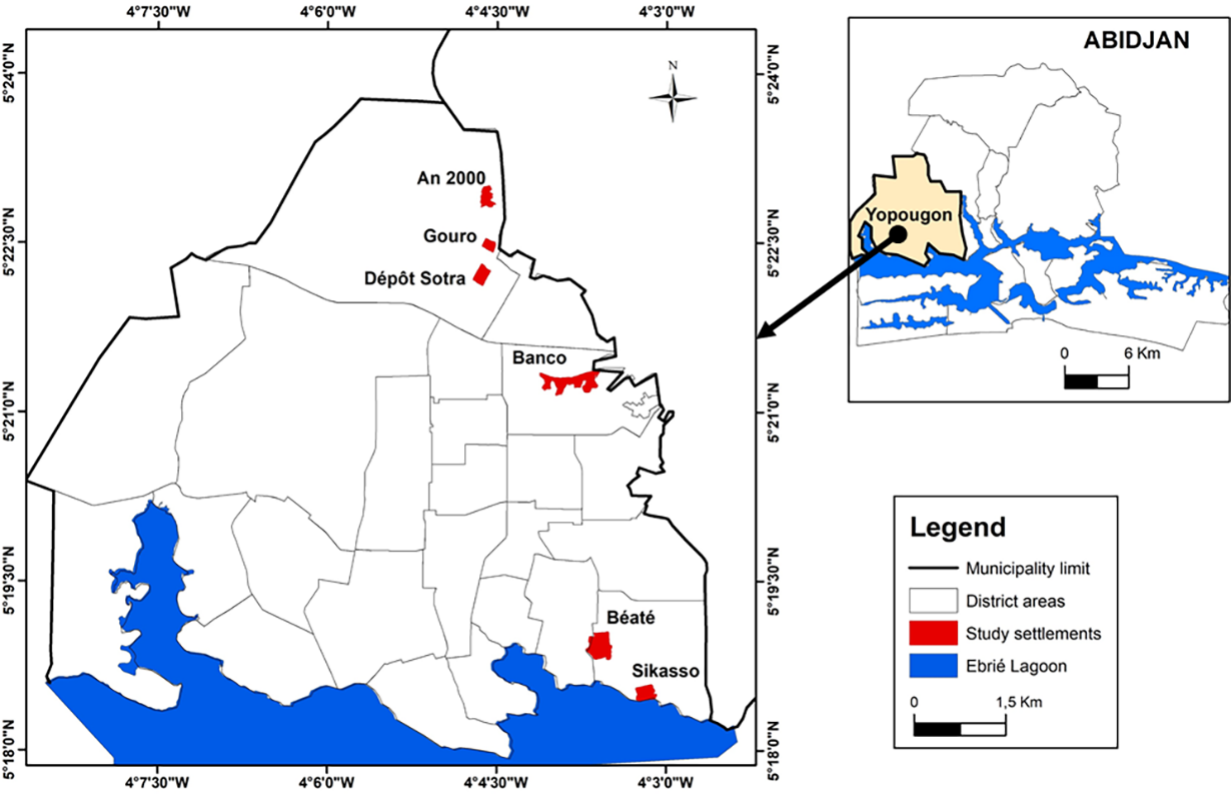
Municipality of Yopougon, Abidjan.

The study was conducted in six poor peri-urban settlements located in the municipality of Yopougon (Fig 1) that were chosen based on the size of the settlement (i.e. small size: from 150 to 200 households), the level of homogeneity between the settlements and risky practices previously observed with regard to water and sanitation (e.g. open defecation and unregulated waste disposal). The six settlements investigated were An 2000, Banco, Béaté cocoterie, Dépôt Sotra, Gouro and Sikasso. These settlements are characterised by:

**An 2000:** The settlement An 2000 is more formal than the other settings investigated because it was developed by authorities to provide accommodation for poor populations evicted from informal settlements at risk of flood or landslides. This settlement is close to the road and residents are connected to the water supply system and have access to basic sanitation. However, because of the rapid population growth, new residents settled at the outskirts of this settlement in traditional house called ‘cicobois’ without latrines and practicing open defecation.
**Gouro and Dépôt Sotra:** These two settlements are unplanned areas without adequate sanitation and water supply system.
**Banco, Béaté cocoterie and Sikasso:** these three settlements are villages located in an urban setting.

The map of the study area was obtained through cartography and GIS investigations. GPS coordinates of the selected settlements were recorded during field visits and were integrated into the existing digital map database available at the GIS and Remote Sensing Unit (CURAT) of the university. These investigations allowed elaborating the map.

### Data collection

A cross-sectional survey was conducted in June 2015 in 556 households that were randomly selected. The sample size was calculated according to equation 1 defined by the World Health Organisation [15]:

N=P*Q[E/L]2 (1)

N, minimum sample size; P, estimate of the expected proportion (prevalence); Q, the value of (1-P); E, margin of error tolerated (statistical risk in %); L, critical value (1.96 for the risk 5%).

The value of the prevalence was assumed to be 50%. The application of equation 1 with a precision of 5% and a 95% confidence level resulted in a minimum sample size of 384 households. To enhance the representativeness of the sample, it was planned to enhance the sample size to 600 households (with 100 households per settlement); however, finally this numbers was reduced to 556 households surveyed due to some non-responses.

A pilot survey was conducted to test the validity and applicability of the questionnaire and to help refining the questionnaire. The final questionnaire was divided into several sections including basic information on the socio-demographic characteristics (e.g. gender, age, marital status, education level and household size) and the WASH conditions (e.g. drinking water supply, wastewater management and sanitation, open defecation, hand washing and pseudo-malaria and diarrhea diseases contracted by the household during the past two years). The questionnaire was administered by six previously trained interviewers. Responses were sought from the head of household or any other representative of the household above 14 years of age. One person per household was interviewed.

### Statistical analysis

The data collected were recorded in EpiData version 3.1 and double-entered by two operators into two different databases. The information was subsequently compared and inconsistencies corrected based on the corresponding questionnaire sheet. The data were analysed using Stata 14IC software [16, 17]. Descriptive analysis was done to determine the proportion of households with access to water and sanitation. Univariate logistic regression was used to assess the independent association between each factor and the outcomes with a statistical significance threshold of 0.2 (p ≤ 0.20) [18]. Multicollinearity was assessed by checking variance inflation factors (VIF). A value of 4 has been considered as a maximum level of VIF [19] and no variable with VIF greater than 4 was found. An interaction term designed as cross-product of religion and education was included in the different models, and likelihood ratio tests were used to estimate the significance of the interaction. The interaction variable did not improve the model significantly, so we have not considered it in the final model.

For each of the two outcomes, access to clean water and access to improved sanitation, multivariate logistic regression in Stata 14IC software was used to fit an explanatory model. These outcomes were defined based on information on improved water and improved sanitation provided by WHO and United Nations Children’s Fund [20] (Table 1).

**Table 1:** Definition of access to drinking-water and improved sanitation according to WHO and UNICEF.

Variables	Definition of WHO and UNICEF	Definition adapted according to the study
Access to drinking water/improved water source	Use the following facilities: • Piped water • Public tap/standpost • Tubewell/borehole • Potected dug well • Protected spring • Rainwater	Households that use in their household only an improved water source (tap water, water reselling or standpipe and hand pump)
Access to improved sanitation	Using basic sanitation facilities that are likely to ensure hygenic separation of human excreta from human contact. They include • Flush/pour flush to: piped sewer, system, septic tank, pit latrine • Ventilated improved pit (VIP) latrine • Pit latrine with slab • Composting toilet	Households with a basic sanitation facility in their household (flush to sewerage or septic tank, improved latrines with ventilated pit, or SanPlat or basic pits with slab tanks), and the destination of faecal sludge is not the street or the environment or the drainage channels.

### Construction of logistic regression models

To build the two logistic regression models, six potential confounding factors collected in the cross-sectional survey and describing the socio-economic characteristics of the study population were identified. These were: (i) the settlements: the location of the residence; (ii) the level of education of the respondent: not educated or highest level achieved (primary, secondary and higher school); (iii) the religion: christianity, islam and ‘other’; (iv) housing condition reflecting the socio-economic status (e.g. very-low class socio-economic households: traditional housing made of wood and plastic, low class socio-economic households: shared courtyard referring to grouped houses with a common courtyard and latrines accomodating several households, and middle class socio-economic households: economic or social housing); (v) household size: number of people living in a household as a continuous variable; and (vi) the presence of the household head’s wife at home during the survey. In poor areas, most of the household head’s wives stay at home for household duties, some of them, however, have an employment and entrust their domestic chores to other people.

The model was not reduced as the number of model parameters does not clearly exceed 10% of the number of cases or non-cases. The odds ratio (OR) and corresponding confidence intervals (CI) were reported.

## Results

### Socio-demographic characteristics of the respondents

In total, 556 households were investigated in the six selected settlements of the municipality of Yopougon. The main socio-demographic characteristics of the respondents based on the frequency analysis, are shown in Table 2. In all the settlements, more than half of the respondents were young and predominantly female. Secondary and high school were the highest level achieved by the majority of the respondents (30.8%) and 47% had no formal education. Also, more than half of the individuals were married and about 30% were single. Islam and christianity were the two most represented religions, except in Sikasso where Islam is practiced at 95%.

**Table 2:** Socio-demographic characteristics of the respondents in the different settlements.

Variables	Item	Percentage frequency							
		An 2000	Banco		Bat	Dépôt Sotra	Gouro	Sikasso	Average
**Sex**	Male	31.7	24	25.2		22.2	20	34.6	25.6
	Female	68.3	76	74.8		77.8	80	65.4	74.4
**Age**	15–20 years	8.9	11	12.2		10.3	13	14.5	11.4
	21–40 years	74.3	67	75.5		76.3	76	56.4	72.1
	41–60 years	13.9	17	12.2		11.3	11	21.8	14
	> 60 years	3	5	0		2.1	0	7.3	2.5
**Education level**	No education	36.6	44.9	41.8		53.5	44.4	72.2	47
	Primary school	20.8	27.5	23.5		24.2	22.2	9.3	22.2
	Secondary/ High school	42.6	27.5	34.7		22.2	33.3	18.5	30.8
**Religion**	Christianity	50.5	51	52		28.3	54	0	42.5
	Islam	42.6	43	40.8		68.7	37	94.5	51.2
	Other	6.9	6	7.1		3	9	5.5	6.3
**Matrimo-nial status**	Married	75.2	65	78.8		62.2	75.8	55.6	69.8
	Divorced	1	1	0		2	2.1	5.6	1.7
	Widower	0	7	0		3.1	1	11.1	3.1
	Single	23.8	27	21.2		32.7	21.1	27.8	25.4
**Presence of wife at home**	Yes	58	53.2	73		68.8	68.5	56.8	63.5
	No	42	46.8	27		31.2	31.5	43.2	36.5

### Water supply

**Fig 2:**
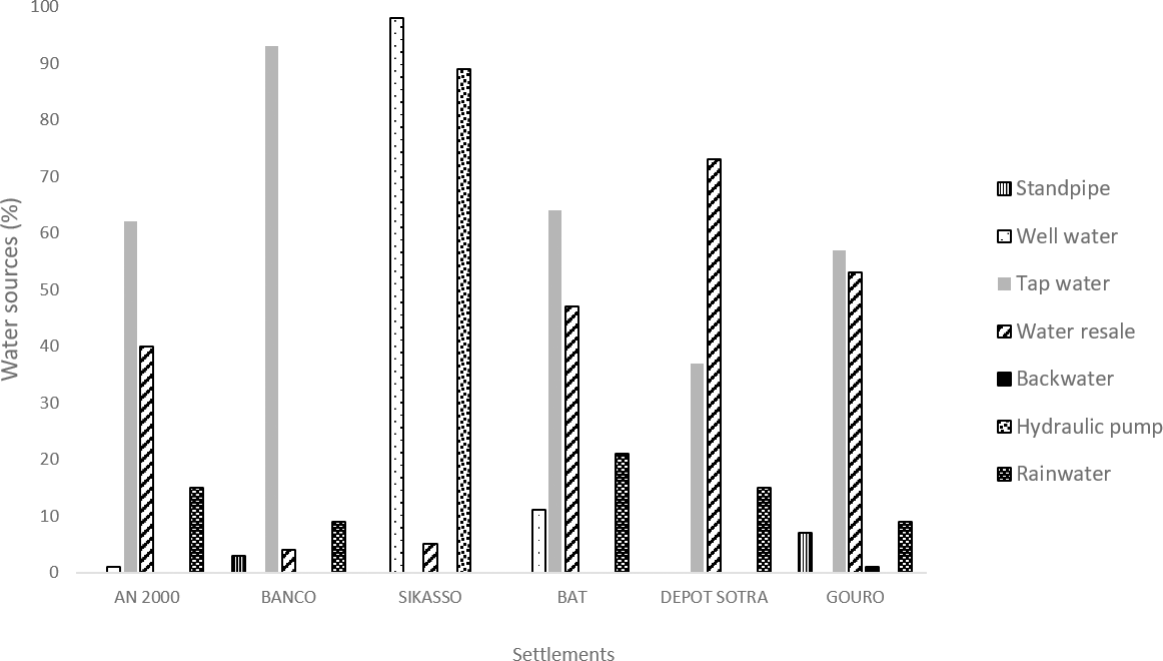
Water supply (%) in each settlement of the study area.

Several types of water sources were used in the different settlements. The main ones were tap water (57%), water for resale (40%), well water (12%), rainwater (12%) and water obtained by hand pump (9%). Details in water supply are highlighted in Fig 2.

### Management of wastewater and excreta

Water used for dishes and laundry was mostly spilled (70%) into the streets in all areas investigated. Eight percent of all households assessed did not have any latrines with household members practicing open defecation. In addition, in 18% of households, content from the latrines was released onto the street contributing to the degradation of the environment and leading to health risks.

### Access to clean water

The results revealed that 424 of the 554 respondents (76%) had access to clean water. According to univariate analysis, households with less access to water were significantly ‘living in a household sized 6–10 people’ (p = 0.07), ‘living in Béaté’ (p = 0.07) or ‘are living in Sikasso’ (p < 0.001) and ‘are practicing a religion other than christianism’ (p ≤ 0.01). In contrast, greater access to water was associated with ‘living in Banco’ (p = 0.15), ‘living in a very low class socio-economic household’ (P = 0.02) and the ‘presence of the household head's wife at home’ (P < 0.01). A total of 166 household head's wives (37%) were not present at home during the survey.

The multivariate logistic regression model also showed that the only factor significantly associated with greater access to clean water was the presence of the household head's wife at home (OR = 3.57; 95% CI: 1.74, 7.31). Several factors were associated with less access to clean water, including ‘living in a low class socio-economic household’ (OR = 0.44; 95% CI: 0.20, 0.97) and ‘residence at the settlements Béaté cocoterie (OR = 0.21; 95% CI: 0.08, 0.55) or Sikasso’ (OR<0.01; 95% CI: <0.01, 0.01). The results of the univariate and multivariate logistic regression model are fully presented in Table 3.

**Table 3:** Univariate and multivariate logistic regression for associations between identified factors and access to clean water in settlements investigated in Yopougon.

	Univariate logistic regression			Multivariate logistic regression		
	OR	[95% CI]	p-Value	OR	[95% CI]	p-value
**Settlement**						
AN 2000	1.00			1.00		
Banco	1.90	[0.79, 4.54]	0.15	1.44	[0.47, 4.45]	0.52
Béaté cocoterie	0.53	[0.26, 1.06]	0.07	**0.21**	**[0.08, 0.55]**	**<0.01**
Dépôt Sotra	1.05	[0.49, 2.27]	0.89	0.78	[0.26, 2.28]	0.64
Gouro	1.69	[0.73, 3.94]	0.22	1.14	[0.40, 3.29]	0.80
Sikasso	<0.01	[<0.01, 0.03]	<0.001	**<0.01**	**[<0.01, 0.01]**	**<0.001**
**Housing condition**						
Middle class socio-economic household	1.00			1.00		
Low class socio-economic household	1.11	[0.72, 1.72]	0.62	**0.44**	**[0.20, 0.97]**	**0.04**
Very low class socio-economic household	2.40	[1.13, 5.08]	0.02	0.75	[0.26, 2.18]	0.59
**Household size**						
1–5	1.00			1.00		
6–10	0.68	[0.44, 1.04]	0.07	0.99	[0.50, 1.98]	0.99
≥11	0.93	[0.49, 1.75]	0.82	1.69	[0.54, 5.24]	0.36
**Religion**						
Christianity	1.00			1.00		
Islam	0.49	[0.32, 0.75]	<0.01	1.07	[0.53, 2.17]	0.85
Other	0.37	[0.17, 0.81]	0.01	0.57	[0.17, 1.86]	0.35
**Education level**						
No education	1.00			1.00		
Primary school	1.04	[0.62, 1.75]	0.87	0.59	[0.28, 1.25]	0.17
Secondary/high school	0.90	[0.57, 1.41]	0.64	1.00	[0.43, 2.32]	0.99
**Presence of wife at home**						
No	1.00			1.00		
Yes	2.01	[1.28, 3.17]	<0.01	**3.57**	**[1.74, 7.31]**	**<0.001**

### Access to improved sanitation

In total, less than half of all investigated households (43%) had access to improved sanitation. Compared with households which had access to improved sanitation those without were significantly associated with ‘living in a very low class socio-economic household’ (p = 0.2), ‘living in all the studied settlements, except An 2000’ (p < 0.001), ‘living in a household sized greater than 11 people’ (p = 0.05), and ‘practicing islam’ (p < 0.01). A ‘higher education level’ was associatied with increased access to improved sanitation (p < 0.001).

In the multivariate logistic regression model, several factors were associated with lower access to improved sanitation. They included ‘living in a very low class socio-economic household’ (OR = 0.37; 95% CI: 0.1, 0.77) and also ‘living in all the studied settlements, except An 2000’ ([0.11<OR<0.39]; 95% CI: 0.05, 0.79). More details regarding univariate analysis and multivariate logistic regression model of the association between identified factors and access to improved sanitation are highlighted in Table 4.

**Table 4:** Univariate and multivariate logistic regression for associations between identified factors and access to improved sanitation in settlements investigated in Yopougon.

	Univariate logistic regression			Multivariate logistic regression		
Caracteristics	OR	[95% CI]	p-Value	OR	95% CI	p-Value
**Settlement**						
An 2000	1.00			1.00		
Banco	0.10	[0.05, 0.20]	<0.001	**0.11**	**[0.05, 0.22]**	**<0.001**
Béaté cocoterie	0.23	[0.13, 0.43]	<0.001	**0.22**	**[0.11, 0.46]**	**<0.001**
Dépôt Sotra	0.15	[0.08, 0.29]	<0.001	**0.20**	**[0.09, 0.42]**	**<0.001**
Gouro	0.39	[0.21, 0.71]	<0.001	**0.39**	**[0.19, 0.79]**	**<0.01**
Sikasso						
**Housing condition**						
Middle class socio-economic household				1.00		
Low class socio-economic household	1.16	[0.79, 1.70]	0.45	1.00	[0.58, 1.73]	0.99
Very low class socio-economic household	0.68	[0.38, 1.22]	0.20	**0.37**	**[0.17, 0.77]**	**<0.01**
**Household size**						
1–5	1.00			1.00		
6–10	0.80	[0.55, 1.15]	0.23	0.84	[0.51, 1.39]	0.50
≥11	0.59	[0.34, 1.01]	0.05	0.94	[0.44, 2.01]	0.88
**Religion**						
Christianity	1.00			1.00		
Islam	0.39	[0.27, 0.57]	<0.001	0.71	[0.43, 1.16]	0.17
Other	0.72	[0.35, 1.47]	0.37	0.77	[0.29, 2.08]	0.61
**Education level**						
No education	1.00			1.00		
Primary school	1.22	[0.78, 1.92]	0.38	1.01	[0.58, 1.78]	0.96
Secondary/high school	2.60	[1.74, 3.89]	<0.001	1.66	[0.91, 3.02]	0.10
**Presence of wife at home**	** **					
No	1.00			1.00		
Yes	1.18	[0.80, 1.76]	0.40	1.41	[0.84, 2.37]	0.19

## Discussion

In the investigated settlements, the proportion of the type of water source used, varied significantly from one settlement to another (p < 0.001). Several sources of clean drinking water were identified (i.e. water for resale, tap, hydraulic or hand pump, standpipe). However, the technology and accessibility of the sources for improved drinking water are often inadequate, resulting in pressure drops and untimely water shortages, hence people tend to use unimproved water sources including unprotected wells. The results of the study showed that more than one fifth of households in the investigated settlements lack access to clean water. Sikasso and Béaté cocoterie are settlements where households have lower access to clean water compared with those who live in other investigated settlements. Originally, these settlements are villages with wells for water supply. In Béaté cocoterie, 11% of surveyed households still using unprotected wells because of recurrent water shortages. In Sikasso, the situation was found to be somewhat different. Households were not connected to the drinking water system and there was only one hand pump for the supply of this settlement. People have to pay to access it and, moreover, a significant physical effort is involved in operating the pump. Almost all people surveyed therefore stated to prefer using well water.

‘Living in a low class socio-economic household’ was also associated with lower access to clean water. Higher socio-economic classes are more likely to access improved water because they have the ability to purchase private alternative sources during shortages [21]. The presence of the household head's wife at home (OR = 3.57; 95% CI: 1.74, 7.31) was significantly associated with greater access to clean water. In Africa, women and girls spend more time fetching water compared to men and boys. Highlights of previous study conducted in 1994 in SSA [22] are still relevant today. This previous study reported that women spent more than 200 to 700 hours a day fetching water. Water is a main ingredient in food processing and other major household and market economies in which women in Africa are mostly engaged. In the African context, most of women’s tasks in the household such as cooking, washing, drinking and child-rearing, rely considerably on water and sanitation services [23] [24]. Therefore, in this condition, the limited access to water and sanitation, will not only exacerbate women’s and girls’ time and labor burden, it will also affect their livelihoods [22] [25]. The importance of women in WASH provision does not mean that women should be encouraged to stay at home, but they should be involved at all levels of programming for more sustainability of WASH projects as highlighted in previous research. This study suggests giving leadership to women in water and sanitation planning and implementation, since they are most knowledgeable and most affected by these services. [26].

Surprisingly, our multivariable analysis did not identify the level of education as a risk factor for lacking access to drinking water. This differs from a recent study that reported a significant association between access to improved water sources for domestic uses and the level of education in low-income urban areas of Accra [21]. Higher educational achievement of an individual would mean more opportunities to get good salaried employment and enough financial ressources for greater access to improved water. Nowadays, because of the increase in the unemployment rate, higher educational achievement does not ensure a well-paid job. This result reinforces the idea of the important role of women in access to improved water, independently of their education level. In the study area, 60% of the wives who stayed at home had received no formal education.

Multivariate analysis showed that household size was not significantly associated with improved water. Generally, bigger household size means huge water needs and more expenses. Household leaving in this condition could use unimproved water which are free of charge. Previous studies showed that in precarious settlements the majority of households used water reselling more expensive than domestic tap water [10].

Moreover, wastewater management in the disadvantaged areas of Yopougon is generally inadequate. Almost three quarters of all surveyed households reported to discharge their wastewater onto the street (nature and draining channel). Previous studies carried out in 2004 in precarious areas of this municipality revealed that those same practices lead to the emergence of diseases [13], suggesting that more than 10 years later, there is still much to be done for a better wastewater management. The urban sanitation master plan of Abidjan has not been updated since it was created in 2000 [27]. It has consistently failed to improve sanitation in Abidjan because of the accelerated population growth and the rapid urbanization rate [13]. In addition, this plan does not cover the underprivileged areas, leaving them without adequate sanitation system [28].

In Yopougon, there are three main actors in the management of sanitation, namely SODECI, the National Office of Sanitation and Drainage (French abbreviation: ONAD), representative of the state and the Municipality. SODECI takes care of the maintenance of the sewerage system, while ONAD is responsible for ensuring access to adequate sanitation in rural and urban areas (formal neighborhoods). The municipality monitors the collective network and the backflows and handles complaints. The complaints about problems of backflows are transmitted to SODECI and ONAD, as the Muncipality lacks the resources to cope with difficulties in this area itself (Interview with one representative of the Municipality, 2015). In poor peri-urban settlements of Yopougon, particularly in those studied, there are two broad types of sanitation systems. The more popular on-site sanitation includes septic tanks and cesspools is used by nine out of ten households, while the remaining households rely on collective sanitation which is organized through private household connections to the city sewer collection network.

Almost half of the households investigated reported access to improved sanitation, approximately 10% of households in these neighborhoods practiced open defecation and 20% rejected the products of the latrines in the environment. As for access to clean water, there were disparities in access to improved sanitation that can be associated with several factors. The settlement was the main factor associated with lower access to improved sanitation. Compared with the settlement An 2000, all other investigated areas were less likely to have access to improved sanitation. The likely reason is that An 2000 is close to the road compared with the other neighborhoods, making it easier for example the coming of fecal sludge emptying trucks into the area. As in rural areas, access to improved water and sanitation in poor peri-urban settlements may be much lower within inhabitants of remote or difficult areas of access compared with those of areas that are easier to reach [11]. In addition, in this settlement most of households that are currently living there were previously re-settled by the government from different shantytowns presenting risks of flood or landslides. That is why they had access to a minimum of basic infrastructure compared with those living in other poor peri-urban areas. However, in general, in the planning of African cities, the need for water and sanitation in poor communities is not adequately taken into account [21, 29]. ‘Living in a a very low class socio-economic household, was also associated with reduced access to improved sanitation (OR = 0.37), confirming that the level of wealth influences the access to improved sanitation. Previous study has stated on this situation making an evidence of a strong link between household wealth and access to improved sanitation [11].

More than half of the population in the poor peri-urban areas studied have a good level of education. This situation is almost the same at the national level as highlighted by the national institute of statistics (INS) [14]. Education level therefore seems not to be an important factor to include for better success in actions for improvements of access to safe water and sanitation as highlighted in this study. However, there is a large representation of religious communities in the investigated settlements, particularly Islam and Christianity. Therefore, successful interventions in these areas should be based on the integration of Muslim and Christian communities. Recent study showed the important role of religious communities for protecting the environment [30], supporting this conclusion.

In the present study, it was not possible to directly explore associations between income and access to clean water or improved sanitation due to the absence of the variables of the household income; however, we used the type of housing as a socio-economic status based on the literature [31].

## Conclusions

Access to improved water and sanitation remains a challenge for poor urban population of Sub-Saharan African cities, particularly those living in poor peri-urban areas. innovative planning approaches tailored to communities’ conditions and based on social context of each sepecific settlement are needed for faster progress in these areas in order to achieve access to basic drinking water and sanitation by 2030 as recommended by the United Nations Sustainable Development Goals (UN-SDG). Those approaches should involve not only local actors and administrative authorities but also religious communities e.g. for WASH communication because of their large representation, and women owing to their important role in ensuring access to clean water in these specific environments.
